# Percutaneous Image-Guided Biopsy for Non-Mass-Forming Isolated Splenomegaly and Suspected Malignant Lymphoma

**DOI:** 10.1371/journal.pone.0111657

**Published:** 2014-11-03

**Authors:** Hiroyuki Tokue, Satoshi Hirasawa, Hideo Morita, Yoshinori Koyma, Masaya Miyazaki, Kei Shibuya, Azusa Tokue, Sachiko Nakano, Yoshito Tsushima

**Affiliations:** 1 Department of Diagnostic and Interventional Radiology, Gunma University Hospital, Gunma, Japan; 2 Department of Radiology, Maebashi Red Cross Hospital, Gunma, Japan; West Virginia University School of Medicine, United States of America

## Abstract

**Background:**

The aim of this study was to evaluate the accuracy, safety, and role of splenic biopsy in the management of patients with non-mass-forming isolated splenomegaly and suspected malignant lymphoma.

**Methods:**

Between 2001 and 2013, 137 biopsies were performed under computed tomography (CT) fluoroscopic guidance in 39 patients. All patients had splenomegaly based on the CT findings and a suspected diagnosis of malignant lymphoma based on their clinical symptoms. The spleen was the only accessible site to perform a biopsy, and no mass lesions could be identified in the spleen.

**Results:**

The overall sensitivity, specificity, and diagnostic accuracy of image-guided biopsy for malignant lymphoma were 88%, 100% and 92%, respectively. Major complications occurred in 3 patients. In 1 patient, transcatheter arterial embolization was performed due to hemorrhage, and two patients needed blood transfusion because of hematoma development, without the need for further treatment.

**Conclusions:**

Image-guided splenic core-needle biopsy is a safe and accurate technique with a high diagnostic accuracy in most patients who with non-mass-forming isolated splenomegaly and suspected underlying malignant lymphoma.

## Background

The incidental finding of isolated splenomegaly (i.e., without the presence of enlarged lymph nodes) during the clinical assessment of patients evaluated for unrelated causes has become increasingly frequent because of the widespread use of imaging techniques.

Splenomegaly may be a manifestation of underlying primary disease or associated disease or previously undiagnosed illness. A tailored diagnostic approach should be used for patients with undiagnosed splenomegaly, and in particular, the presence of neoplasms should be assessed in cases of isolated splenomegaly. However, it is often difficult to diagnose malignant lymphoma in cases of non-mass-forming isolated splenomegaly.

Historically, image-guided percutaneous biopsy of the spleen has been attempted with caution by radiologists because of concerns regarding accessibility and the risk of hemorrhage. In the 1980s, an early report showed a high rate of major complications (13%) for percutaneous biopsy of the spleen performed using a 14-gauge needle [1]. Several more recent publications have reported much lower complication rates on using needles with smaller diameters (18-gauge or less) [2]–[4]. At our institution, percutaneous biopsy is often requested in cases where the spleen is the only accessible site for biopsy. To our knowledge, reports of percutaneous biopsy for the diagnosis of non-mass-forming isolated splenomegaly are incomplete or poor quality.

The aim of this study was to evaluate the accuracy, safety, and role of splenic biopsy in the management of patients who present with non-mass-forming isolated splenomegaly and suspected underlying malignant lymphoma.

## Methods

### Ethics statement

The study was approved by our institutional review board of the “ethics committee of Gunma university hospital” before undergoing the study procedures. Prior to the procedure, a written informed consent was obtained from all patients.

All image-guide splenic interventions performed from 2001 from 2013 at our institution were retrospectively reviewed by using radiology department patient registries and electric medical records. A text query for the words “spleen” or “splenic” on all inteventional readiology reports yielded 151 patients for that period. Patients undergoing splenic artery embolization, splenic fluid aspiration or perctaneous drainage and those with lesions not involving the splenic parenchyma (ie, hilar or perisplenic masses) were excluded from the study. Final analysis revealed 137 splenic biopsies performed in 39 patients.

The medical records of 39 patients (men/women: 21/18, age: 61 years, range: 34–78) were retrospectively reviewed. All patients underwent abdominal CT examination before biopsy. All patients had splenomegaly as diagnosed by CT, and malignant lymphoma was suspected on the basis of their clinical symptoms. In all cases, the spleen was the only accessible site to perform a biopsy, and there were no mass lesions in the spleen. In all patients, the blood culture findings failed to establish a diagnosis. A platelet count of >95000/µL, a prothrombin time of <20 seconds, and prothrombin activity >50% were required for inclusion in the study. The study was approved by our institutional review board, and informed consent was obtained from all patients before undergoing the procedures.

After we reviewed the diagnostic abdominal CT findings, we placed the patient in a prone or supine position. After skin preparation and under local anesthesia, core needle biopsy using a Temno 18-gauge cutting-needle (Carefusion, McGaw Park [IL], USA) was performed for histological examination under intermittent CT fluoroscopic guidance (Aquilion 64; Toshiba, Tokyo, Japan). For multiple biopsies, different points in the spleen were sampled.

CT was performed to exclude the presence of bleeding, immediately after the biopsy and as a follow-up examination within 24 hours, as required. A biopsy was considered successful if it provided sufficient material for pathologic diagnosis.

The patients’ clinical information and final pathology report were reviewed to analyze the diagnostic accuracy of biopsy and related complications. In the presence of high clinical suspicion of lymphoma, if the biopsy result was negative for lymphoma, a decision was made by a team including a hematologist, a pathologist, and a radiologist to repeat the biopsy. The final diagnosis was established via pathological examination of the splenectomy specimen, biopsy of another site, or a combination of the clinical course and imaging follow-up for a minimum of 18 months.

The sensitivity, specificity, and diagnostic accuracy were also calculated for patients, with subsequent follow-up data on the basis of clinical and imaging follow-up and review of medical records for at least 18 months after biopsy. The pathologic results of needle biopsy and surgery were reviewed and divided into diagnostic categories that included lymphoma, and not lymphoma. A designation of a lymphoma lesion was considered a positive result. A positive biopsy result was considered to be true-positive when there was surgical confirmation, when the results of biopsy of another site revealed lymphoma with the same histologic characteristics or when response to chemotherapy was effective. A positive biopsy result was considered to be false-positive when there was no evidence of lymphoma at surgical resection without preoperative chemotherapy or when suspected lesion of lymphoma showed regression at follow-up CT in the absence of therapy. Designation of a not lymphoma lesion was considered a negative result. A negative biopsy result was considered to be true-negative when there was a definitive diagnosis of a benign entity, such as splenitis, or when the suspected lesion of lymphoma subsequently disappeared or decreased in size at follow-up CT. A negative result was considered to be false-negative when there was surgical resection with a diagnosis of lymphoma or when the results of biopsy of another site revealed lymphoma.

All complications were recorded and classified as major or minor according to the guidelines of the Standards of Practice Committee. Major complications were defined as those necessitating major therapeutic intervention, those requiring an unplanned increase in the level of care or prolonged hospitalization (>48 hours), and those resulting in permanent adverse sequelae or death. Minor complications were defined as those requiring no or minimal therapy, including overnight admission for observation only.

## Results

During the study period, 137 biopsies were performed in 39 patients. Each patient in our series underwent 2–5 biopsies. The average number of biopsies was 3.5 per patient. The biopsy technical success rate was 100%, and the results are shown in Table 1. 5 patients underwent surgery, 5 patients underwent biopsy of another site, and 29 patients underwent clinical observation. Malignant lymphoma was diagnosed on the basis of splenic cutting-needle biopsy findings in 21 patients. In 18 patients with malignant lymphoma, immunohistochemical staining was performed and allowed subtype classification of lymphoma. The overall sensitivity, specificity, and diagnostic accuracy of image-guided biopsy for malignant lymphoma were 88% (21 of 24 cases), 100% (15 of 15 cases), and 92% (36 of 39 cases), respectively (Table S1).

The complication types and frequencies are provided in Table S2. Major and minor complications occurred in 3 (8%) and 17 patients (44%), respectively.

Ten patients experienced transient pain that was manageable with local anesthesia. Ten patients developed bleeding complications; these were resolved in 7 patients with conservative therapy alone whereas 2 patients required blood transfusion because of hematoma development, without the need for further treatment.

**Figure 1:**
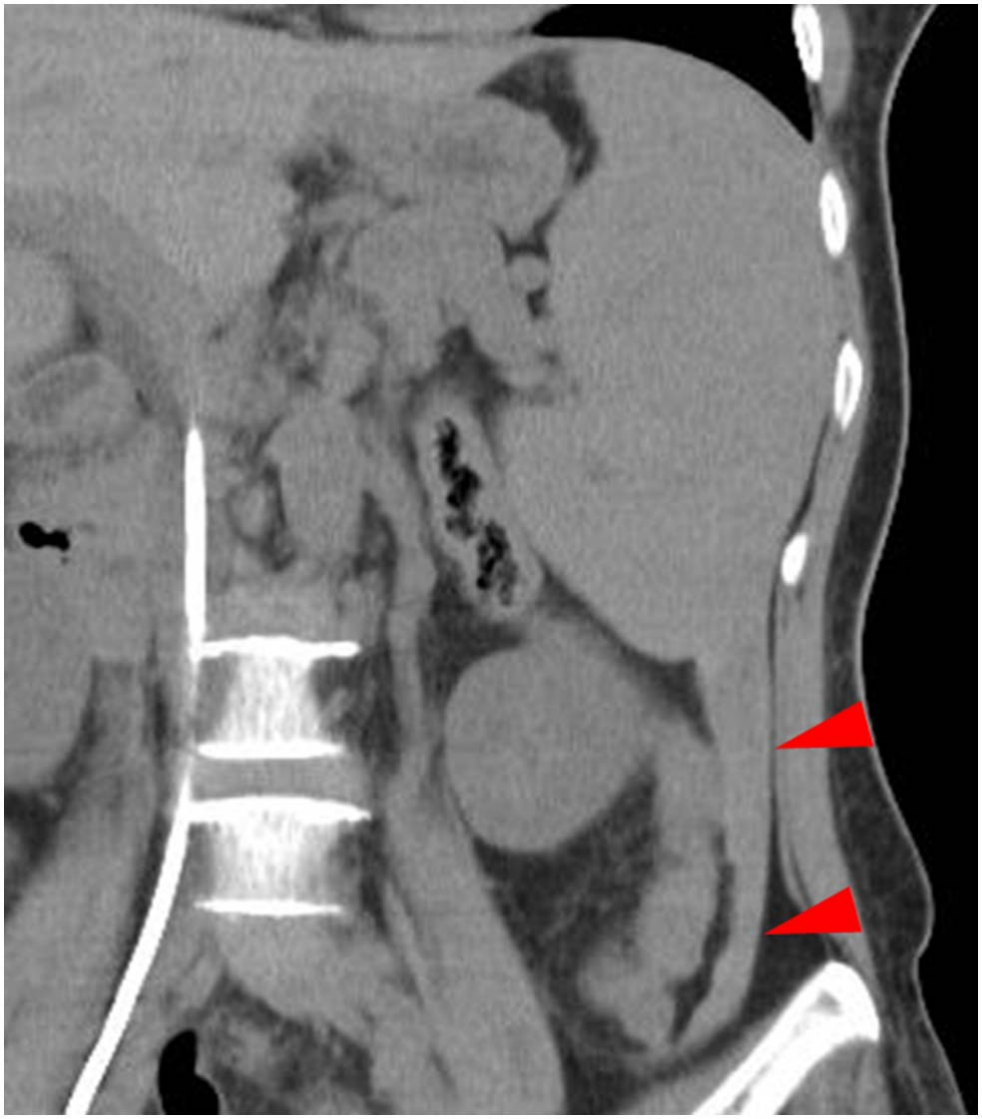
A 55-year-old woman with anemia underwent splenic biopsy. Immediately after biopsy, no obvious hematoma was observed around the spleen; however, mild hematoma formation was seen in the paracolic gutter (arrowheads).

**Figure 2:**
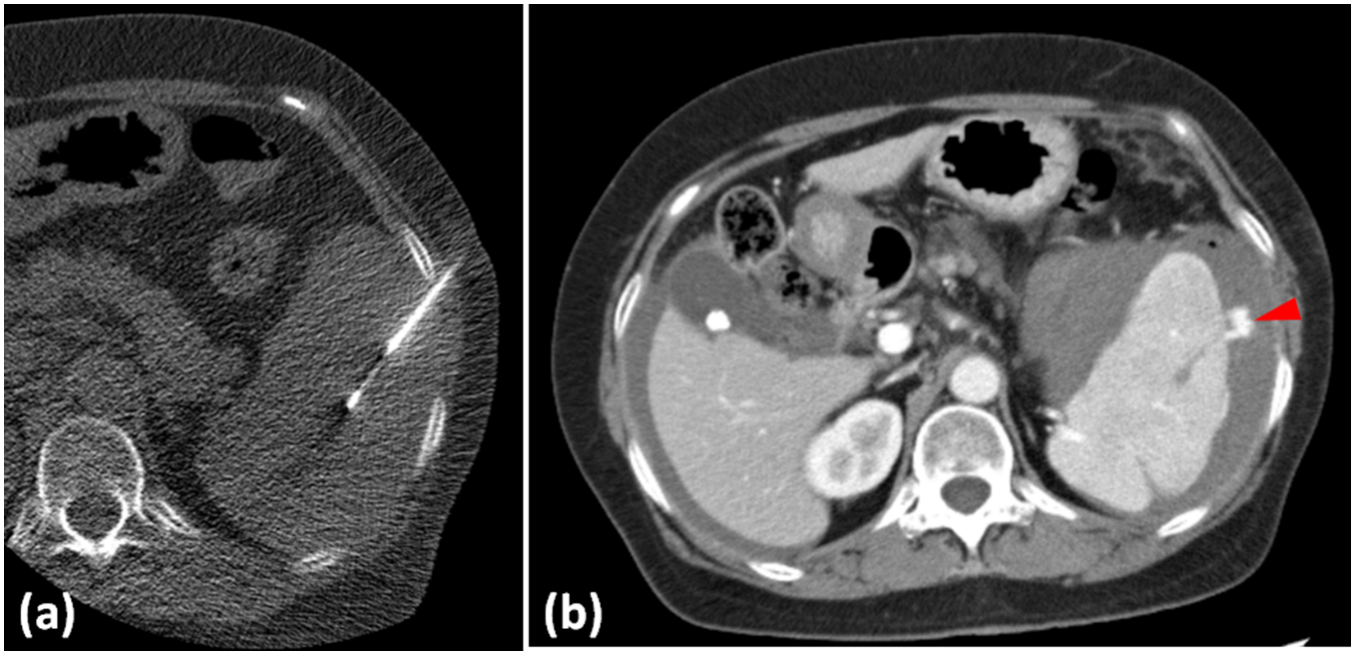
A 64-year-old woman with pancytopenia underwent splenic biopsy. (a) Splenic biopsy was performed under computed tomography (CT) guidance. (b) Immediately after biopsy, contrast-enhanced CT showed a peritoneal hematoma and extravasation around the spleen (arrowhead). Transcatheter arterial embolization was immediately performed.

In 2 patients, the computed tomography (CT) findings revealed mild hemorrhage around the spleen immediately after biopsy. In 7 patients, no obvious hematoma was observed around the spleen; however, mild hematoma formation was seen in the paracolic gutter (Figure 1). In 1 patient with active bleeding immediately after biopsy, transcatheter arterial embolization was performed (Figure 2).

## Discussion

In cases where splenomegaly is the only abnormal finding, the spleen is the most accessible organ for biopsy, and tissue diagnosis is required, image-guided percutaneous biopsy of the spleen may be a safe procedure with a high diagnostic accuracy, as suggested by our study findings.

Isolated splenomegaly is not uncommon and can be particularly challenging to manage. The risks of serious underlying disease must be balanced against the risk of invasive investigations such as splenic biopsy and diagnostic splenectomy. There are various serious causes of splenomegaly, and malignant lymphoma is an important one.

Historically, percutaneous image-guided procedures have not been widely performed in the spleen because of the perceived increased risk of complications. A widely held view regarding splenic interventions among physicians is that there is high risk of morbidity due to hemorrhage [5].

Although invasive, splenectomy is sometimes useful as a purely diagnostic procedure. However, it is associated with an increased risk of sepsis due to encapsulated organisms (such as *Streptococcus pneumoniae* and *Haemophilus influenzae*) and an increase in blood leukocytes [6]. Additionally, the post-splenectomy platelet count may rise to abnormally high levels (thrombocytosis), leading to an increased risk of potentially fatal clot formation [6]. Therefore, splenectomy may not be suitable for patients with malignant lymphoma.

Two techniques have been described for biopsy of the spleen: fine-needle aspiration (FNA) and core-needle biopsy. The sensitivity and specificity of splenic FNA have not been reported frequently because of the limited number of patients undergoing a further biopsy, diagnostic splenectomy, or post-mortem examination to establish whether FNA provided an accurate diagnosis. Overall, splenic FNA seems more useful in patients with focal splenic lesions, where malignancy can easily be distinguished from benign disease, but much less helpful in the investigation of undiagnosed diffuse splenomegaly. Positive FNA results can suggest and sometimes confirm a diagnosis, but a negative result clearly does not exclude the presence of underlying pathology. Any non-diagnostic, suspicious, or negative biopsy finding should be verified by another diagnostic procedure, particularly if lymphoma is clinically suspected [7].

Splenic core-needle biopsy has been used more recently [1], [2], [9]–[11]. Image-guided percutaneous core-needle biopsy of the spleen demonstrates high diagnostic accuracy and major complication rates, similar to that reported for the liver and kidney, on using 18-gauge needles or those with smaller diameters [12].

Core-needle biopsy has been used to diagnose a wide range of infectious, benign, and malignant conditions. Specimens are adequate for diagnosis in up to 90% of cases, often permitting subtyping of lymphomas, but false-negative results do still occur [9]–[11]. An Italian study found a similar overall diagnostic accuracy for splenic core-needle biopsy and FNA (88.3% and 84.9%, respectively), but better accuracy for the diagnosis of lymphoma with core-needle biopsy than with FNA (90.9% and 68.5%) [2].

Tables S3 and S4 show the diagnostic accuracy and complication rates of splenic biopsy in previous studies included in our review. Compared with these studies, we achieved a higher diagnostic accuracy in our study. This result can be attributed to the high average number of biopsies performed per patient compared with these studies. Our minor complication rate is slightly higher than that in previous studies. In almost all previous reports, image-guided biopsy was performed under the guidance of ultrasonography (US) [1]–[4], [8]–[10], [13]–[19], whereas, in our study, we used CT for image-guidance. In our cases, hematomas did not always accumulate around the spleen after biopsy. These may be overlooked on using US alone, but complications can be detected early on using CT. Therefore, in our study, minor complications may have been over-detected because of the use of CT.

Our study has many limitations, firstly, including the small number of patients, insufficient long-term follow up, the retrospective design, and the inclusion of many patients with previously suspected malignant lymphoma. Secondly, all benign biopsy results were not proven by surgical procedure. Therefore, a selection bias may exist. Clearly, more prospective study data are needed to establish the eligibility criteria for core-needle biopsy among patients with isolated splenomegaly.

## Conclusion

Image-guided splenic core-needle biopsy is a safe and accurate technique with a high diagnostic accuracy in most patients who present with non-mass-forming isolated splenomegaly and suspected malignant lymphoma.

## Supporting Information

Table S1 Splenic biopsy results and final diagnosis in 39 patients. Lymphoma was finally diagnosed in 24 patients with 21 true-positive and 3 false-negative findings for an overall sensitivity of 88% (21/24). Not lymphoma was finally diagnosed in 15 patients with no false-positive and 15 true negative results, for a specificity of 100% (15/15).
(DOCX)

Table S2 Summary of complications in 39 patients who underwent splenic biopsy.
(DOCX)

Table S3 Summary data of the diagnostic accuracy of splenic biopsy.
(DOCX)

Table S4 Summary data on complication rates of splenic biopsy.
(DOCX)
