# Supplementary material for: Percutaneous Image-Guided Biopsy for Non-Mass-Forming Isolated Splenomegaly and Suspected Malignant Lymphoma
Source: PLoS One. 2014 Nov 3;9(11):e111657. doi: 10.1371/journal.pone.0111657 (PMC4218790; doi:10.1371/journal.pone.0111657)
Supplement: Table S1 — Splenic biopsy results and final diagnosis in 39 patients. Lymphoma was finally diagnosed in 24 patients with 21 true-positive and 3 false-negative findings for an overall sensitivity of 88% (21/24). Not lymphoma was finally diagnosed in 15 patients with no false-positive and 15 true negative results, for a specificity of 100% (15/15). (DOCX) [file pone.0111657.s001.docx]

Table S1: Splenic biopsy results and final diagnosis in 39 patients

| Biopsy results | Final diagnosis | No. of patients |
| --- | --- | --- |
| Lymphoma (N = 21) |  |  |
|  | Non-Hodgkin lymphoma | 17 |
|  | Hodgkin lymphoma | 4 |
| Non lymphoma (N = 18) |  |  |
|  | Normal spleen | 4 |
|  | Chronic lymphocytic Leukemia | 3 |
|  | Splenitis | 3 |
|  | Extramedullary Hematopoiesis | 3 |
|  | Non-Hodgkin lymphoma | 2 |
|  | Intravascular lymphoma | 1 |
|  | Still disease | 1 |
|  | Sweet disease | 1 |

Lymphoma was finally diagnosed in 24 patients with 21 true-positive and 3 false-negative findings for an overall sensitivity of 88% (21/24).

Not lymphoma was finally diagnosed in 15 patients with no false-positive and 15 true negative results, for a specificity of 100% (15/15).
