# Supplementary material for: Percutaneous Image-Guided Biopsy for Non-Mass-Forming Isolated Splenomegaly and Suspected Malignant Lymphoma
Source: PLoS One. 2014 Nov 3;9(11):e111657. doi: 10.1371/journal.pone.0111657 (PMC4218790; doi:10.1371/journal.pone.0111657)
Supplement: Table S2 — Summary of complications in 39 patients who underwent splenic biopsy. (DOCX) [file pone.0111657.s002.docx]

Table S2: Summary of complications in 39 patients who underwent splenic biopsy

| Complication | No. of cases |
| --- | --- |
| Hemorrhage requiring TAE | 1 (3%) |
| Hemorrhage requiring transfusion | 2 (5%) |
| Hematoma (resolved with conservative therapy) | 7 (20%) |
| Transient pain | 10 (26%) |

TAE: transcatheter arterial embolization
