# Supplementary material for: Percutaneous Image-Guided Biopsy for Non-Mass-Forming Isolated Splenomegaly and Suspected Malignant Lymphoma
Source: PLoS One. 2014 Nov 3;9(11):e111657. doi: 10.1371/journal.pone.0111657 (PMC4218790; doi:10.1371/journal.pone.0111657)
Supplement: Table S3 — Summary data of the diagnostic accuracy of splenic biopsy. (DOCX) [file pone.0111657.s003.docx]

Table S3: Summary data of the diagnostic accuracy of splenic biopsy

| First author | No. of patients | True positive | False negative | True negative | False positive | Excluded cases | Accuracy　(%) |
| --- | --- | --- | --- | --- | --- | --- | --- |
| Liang [13] | 42 | 19 | 3 | 17 | 3 | 0 | 86 |
| Tam [3] | 156 | 86 | 17 | 36 | 5 | 12 | 85 |
| Gómez-Rubio [4] | 62 | 49 | 7 | 6 | 0 | 0 | 89 |
| Civardi [2] | 398 | 112 | 12 | 250 | 2 | 22 | 91 |
| Muraca [9] | 30 | 21 | 4 | 4 | 1 | 0 | 83 |
| Tokue (present) | 39 | 21 | 3 | 15 | 0 | 0 | 92 |
