# Supplementary material for: Percutaneous Image-Guided Biopsy for Non-Mass-Forming Isolated Splenomegaly and Suspected Malignant Lymphoma
Source: PLoS One. 2014 Nov 3;9(11):e111657. doi: 10.1371/journal.pone.0111657 (PMC4218790; doi:10.1371/journal.pone.0111657)
Supplement: Table S4 — Summary data on complication rates of splenic biopsy. (DOCX) [file pone.0111657.s004.docx]

|  | No. of | No. of |  | Imaging modality | Complications |  |
| --- | --- | --- | --- | --- | --- | --- |
| First author | patients | biopsies | Procedure: needle gauge | used for guidance | Major (No. of patients) | Minor (No. of patients) |
| Lindgren [1] | 32 | 32 | CNB: 14G | US | Hemorrhage requiring splenectomy (1) | Pain (16) |
|  |  |  |  |  | Hemorrhage requiring transfusion (3) |  |
| Suzuki [14] | 8 | 8 | CNB: 21G |  | 0 | 0 |
| Di Stasi [15] | 110 | 160 | FNA: 22G, CNB: 21G | US | 0 | Hematoma (1) |
| Cavanna [16] | 46 | 46 | CNB: 21,22G | US | 0 | 0 |
| Keogan [10] | 43 | 20 | FNA: 22G, CNB: 18,20G | US,CT | 0 | 0 |
| Venkaramu [17] | 35 | 35 | FNA: 22G | US | Hemorrhage requiring transfusion (1) | 0 |
| Civardi [2] | 398 | 453 | FNA: 22G | US | Hemorrhage requiring transfusion (2) | Pain (13) |
|  |  |  | CNB: 20,21G |  | Pneumothorax requiring chest tube (1) | Hematoma (2) |
|  |  |  |  |  |  | Hemoperitoneum (2) |
|  |  |  |  |  |  | Vasovagal episode (1) |
| Muraca [9] | 30 | 81 | CNB: 18,20,21G | US | 0 | 0 |
| Lucey [18] | 23 | 24 | FNA: 20,22,23G | US,CT | Hemorrhage requiring splenectomy (2) | Hematoma (1) |
|  |  |  | CNB: 18,19,20G |  |  |  |
| Lieberman [8] | 20 | 43 | CNB: 20,22G | US,CT | 0 | Hematoma (1) |
| Liang [13] | 42 | 43 | CNB: 18,21G | US | Hemorrhage requiring transfusion (1) | 0 |
| Kang [19] | 74 | 78 | FNA: 22G | US,CT | 0 | 0 |
| Tam [3] | 147 | 156 | FNA: 18,20,22,23G | US,CT | Hemorrhage requiring splenectomy (2) | Pain (15) |
|  |  |  | CNB: 20G |  |  | Hematoma (6) |
|  |  |  |  |  |  | Pneumothorax (2) |
|  |  |  |  |  |  | Hypotension (1) |
| Gómez-Rubio [4] | 52 | 62 | FNA: 20,22,25G | US | Hemorrhage requiring splenectomy (1) | Hematoma (1) |
|  |  |  | CNB: 14,18,19.5G |  |  |  |
| Tokue (present) | 39 | 137 | CNB: 18G | CT | Hemorrhage requiring TAE (1) | Pain (10) |
|  |  |  |  |  | Hemorrhage requiring transfusion (2) | Hematoma (7) |

Table S4: Summary data on complication rates of splenic biopsy

CNB: core needle biopsy, FNA: fine-needle aspiration, G: gauge, US: ultrasonography, CT: computed tomography
